# Investigation of Paraoxonase-1 Genotype and Enzyme-Kinetic Parameters in the Context of Cognitive Impairment in Parkinson’s Disease

**DOI:** 10.3390/antiox12020399

**Published:** 2023-02-07

**Authors:** Boštjan Petrič, Sara Redenšek Trampuž, Vita Dolžan, Milica Gregorič Kramberger, Maja Trošt, Nikola Maraković, Marko Goličnik, Aljoša Bavec

**Affiliations:** 1 Institute of Biochemistry and Molecular Genetics, Faculty of Medicine, University of Ljubljana, 1000 Ljubljana, Slovenia; 2 Department of Neurology, University Medical Center, 1000 Ljubljana, Slovenia; 3 Chair of Neurology, Faculty of Medicine, University of Ljubljana, 1000 Ljubljana, Slovenia; 4 Karolinska Institutet, Department of Neurobiology, Care Sciences and Society (NVS), Division of Clinical Geriatrics, 141 83 Huddinge, Sweden; 5 Institute for Medical Research and Occupational Health, 10000 Zagreb, Croatia

**Keywords:** Parkinson disease, cognitive impairment, paraoxonase 1, paraoxonase activity, single-nucleotide polymorphisms, iFIT

## Abstract

Cognitive impairment is a common non-motor symptom of Parkinson’s disease (PD), which often progresses to PD dementia. PD patients with and without dementia may differ in certain biochemical parameters, which could thus be used as biomarkers for PD dementia. The enzyme paraoxonase 1 (PON1) has previously been investigated as a potential biomarker in the context of other types of dementia. In a cohort of PD patients, we compared a group of 89 patients with cognitive impairment with a group of 118 patients with normal cognition. We determined the kinetic parameters K_m_ and V_max_ for PON1 for the reaction with dihydrocoumarin and the genotype of four single nucleotide polymorphisms in *PON1*. We found that no genotype or kinetic parameter correlated significantly with cognitive impairment in PD patients. However, we observed associations between *PON1* rs662 and PON1 K_m_ (*p* < 10^−10^), between *PON1* rs662 and PON1 V_max_ (*p* = 9.33 × 10^−7^), and between *PON1* rs705379 and PON1 V_max_ (*p* = 2.21 × 10^−10^). The present study is novel in three main aspects. (1) It is the first study to investigate associations between the *PON1* genotype and enzyme kinetics in a large number of subjects. (2) It is the first study to report kinetic parameters of PON1 in a large number of subjects and to use time-concentration progress curves instead of initial velocities to determine K_m_ and V_max_ in a clinical context. (3) It is also the first study to calculate enzyme-kinetic parameters in a clinical context with a new algorithm for data point removal from progress curves, dubbed iFIT. Although our results suggest that in the context of PD, there is no clinically useful correlation between cognitive status on the one hand and PON1 genetic and enzyme-kinetic parameters on the other hand, this should not discourage future investigation into PON1’s potential associations with other types of dementia.

## 1. Introduction

Idiopathic Parkinson’s disease (PD) is the most common parkinsonian syndrome. Although dementia is a defining symptom in some parkinsonisms (e.g., dementia with Lewy bodies), motor symptoms are the cardinal feature of PD. However, non-motor symptoms are nevertheless very important since 22–48% of all PD patients exhibit dementia as a non-motor symptom [1]. PD dementia (PDD) is distinguished from other types of dementia, such as Alzheimer’s disease (AD) and vascular dementia, which may, however, co-occur.

The incidence of cognitive impairment that leads to PDD increases with age and duration of PD. Up to 75% of PD patients will develop cognitive impairment within 10 years of PD onset [1]. The most common pharmacological treatment for PDD consists of acetylcholinesterase inhibitors (e.g., rivastigmine), which were primarily developed for AD but were later shown to be useful in the context of PDD as well. They are also often prescribed to treat visual hallucinations in the context of PD. The other class of drugs for PDD consists of NMDA inhibitors, such as memantine [2]. Nevertheless, similar to other common dementias, PDD is irreversible in most patients [3].

It is known that in the development of PD, oxidative stress plays a large role [4]. In the blood of PD patients, several oxidative-stress-related small molecules, such as homocysteine [5] and coenzyme Q10 [6], as well as enzymes such as superoxide dismutase [7] and xanthine oxidase [8], have been shown to have significantly different concentrations in PD compared to healthy controls. Some of these have also been investigated in relation to the cognition of PD patients, e.g., homocysteine [5,9]. Other serum/plasma factors, such as uric acid [10], epidermal growth factor [11,12], and C-reactive protein [13], have also been associated with PDD. Despite these promising results, there is still no clear biomarker for PDD, and thus the search for better biomarkers continues [5].

Paraoxonase 1 (PON1) is a calcium-dependent enzyme that is present in human blood plasma and associated with high-density lipoprotein particles. Some studies have also investigated PON1 in other body fluids, such as seminal [14,15] and cerebrospinal fluid [16,17]. PON1 is interesting in the context of PD for two main reasons. First, the main function of PON1 in the body is considered to be antioxidative, and oxidative stress has been implicated as a contributing factor to PD [18]. Second, PON1 can hydrolyze organophosphates, which are commonly used as insecticides (e.g., paraoxon), and organophosphate exposure is known to be a potential risk factor for PD [19]. Hence, PON1 has been investigated as a possible protective agent in subjects exposed to organophosphates [20,21].

PON1 can be investigated in several different ways, most notably by (1) genotyping individual single-nucleotide polymorphisms (SNPs) on the *PON1* gene, (2) measuring enzyme concentration (which is usually performed with an enzyme-linked immunosorbent assay, i.e., ELISA), and (3) measuring enzyme activity with an artificial substrate of PON1 [22]. Genetic studies on *PON1* in PD focused on two SNPs that affect the amino-acid sequence: rs662 (Q192R) and rs854560 (L55M). The L55M polymorphism has particularly been associated with the risk of developing PD in several studies. A meta-analysis by Mota et al. reviewed nine studies [23], of which five identified a higher risk of developing PD for 55MM carriers [24,25,26,27,28], whereas four did not find an association [29,30,31,32]. Another study, performed on a population of agricultural workers, found that among subjects with a history of organophosphate exposure, those with a 55MM-192QQ diplotype showed an increased risk of developing PD compared to 55LL-192RR carriers [33].

PON1 enzymatic activity can be measured using different artificial substrates, which belong to three main groups: lactones (lactonase activity), arylesters (arylesterase activity), and organophosphates (aryldialkylphosphatase, more commonly known as paraoxonase activity) [34,35]. Especially the latter two are commonly used to measure enzymatic activity [36]. The three enzymatic activities do not necessarily correlate with one another, and thus one cannot be extrapolated to the other two [34].

PON1 enzymatic activity has also been investigated in the context of PD. One study found decreased paraoxonase and arylesterase activity in PD patients compared to controls [37]. In another study, decreased paraoxonase activity was associated with PD progression [38]. Decreased paraoxonase activity was also correlated with increased oxidative stress, increased lipid peroxidation, and altered iron metabolism biomarkers in the serum/plasma of PD patients [37,39,40]. PON1 enzymatic activity has also been investigated in the context of Alzheimer’s dementia and vascular dementia, where it has been demonstrated that paraoxonase activity and arylesterase activity are both reduced in dementia patients compared to healthy controls [41,42,43,44]. Several other types of dementia, however, have not yet been investigated in connection with PON1; notable among them is PDD.

Some studies indicate that oxidative stress might affect the Michaelis constant (K_m_) of PON1 [45]. In the context of catalase and related oxidoreductases, K_m_ has been shown to be a better indicator of oxidative stress than maximal velocity V_max_; the authors of the study suggest that stress results in post-translational modifications of the enzyme, such as oxidation of thiol groups, which then change the enzyme’s affinity for its substrate [46]. Under high-stress conditions, oxidative damage to PON1 can be caused by its neighbor in high-density lipoprotein particles, the enzyme myeloperoxidase [47]. Of note, high oxidative stress has been proposed to be a contributing factor to PDD [48].

The aim of the present study was to investigate whether PDD is associated with the genotype or kinetic activity of PON1. We evaluated whether cognitive test scores, as determined with the mini mental state examination (MMSE) or the binary division of patients (dementia vs. no dementia), are correlated with any of the above-mentioned enzymatic parameters K_m_ and V_max_. Furthermore, we investigated the associations between different PON1 kinetic parameters and *PON1* genotypes, as well as between the enzyme kinetics and genetics on the one hand and demographic or clinical parameters on the other hand. Finally, we wanted to assess these associations on our own sample of patients in order to demonstrate the accuracy of our newly developed method that we use for data analysis (e.g., iFIT).

## 2. Materials and Methods

### 2.1. Patients

A total of 231 PD patients were recruited from the Neurology Clinic at the University Medical Centre in Ljubljana from October 2016 to April 2018. The study protocol was approved in advance by the Slovenian Ethics Committee for Research in Medicine (0120-268/2016/11 and 0120-296/2016/11). Upon inclusion, all subjects gave written informed consent in accordance with the Declaration of Helsinki. Patient demographic and clinical data were collected during an interview and from the medical documentation; blood samples were also collected. Detailed information about the study cohort is presented in Table 1 and is also available elsewhere [49].

The general inclusion criteria were as follows: (1) a clinical diagnosis of PD, made by a movement disorder specialist in accordance with the UK Parkinson Disease Society Brain Bank criteria [50], (2) absence of secondary or atypical forms of parkinsonism, (3) the availability of all necessary clinical data, (4) ongoing treatment with levodopa or dopamine agonists that began at least 3 months prior to the inclusion.

Patients were divided into a group with dementia and a group without dementia based on their clinical diagnoses and MMSE scores. The patient inclusion criteria for the dementia group were as follows: (1) an MMSE score of less than 26, (2) a prior diagnosis of PDD or unspecified cognitive decline, or (3) antidementive treatment but lack of visual hallucinations. The patient inclusion criteria for the group without dementia were as follows: (1) an MMSE score of 26 or more and (2) a record that did not indicate a cognitive decline. The exclusion criteria for both groups were as follows: (1) other forms of dementia (e.g., AD); (2) no MMSE in the past 2 years; and (3) a combination of MMSE score of 26 or higher, present visual hallucinations, and antidementive treatment (e.g., rivastigmine). The reason behind criterion (3) was that cognitive decline and visual hallucinations are both indications for prescribing antidementives. We used the most recent MMSE results, which were not more than 2 years old.

Patients who could not be classified into any of the two groups were not accounted for when comparing cognitive status with genetic and enzymatic parameters but were accounted for when comparing different enzymatic parameters and/or genetics with each other.

### 2.2. DNA Isolation and Genotyping

Peripheral blood samples were collected into EDTA tubes and immediately centrifuged at 2200× *g* and 4 °C for 10 min. Afterward, the plasma and cellular fractions were stored separately at −80 °C.

Genomic DNA was isolated from white blood cells with the FlexiGene DNA Kit (Qiagen, Hilden, Germany) according to the manufacturer’s protocol. Based on the available literature, four different *PON1* SNPs (rs662, rs854560, rs705379, and rs75381) were included in the analysis. These four common functional SNPs have already been associated with PON1 activity in several previous studies [32,36,51,52,53,54,55]. SNPs were genotyped using the KASPar assay according to the manufacturer’s instructions. Their additional data are displayed in Supplementary Table S1. A total of 10% of all samples was genotyped twice as a form of quality control; the results were the same as the original measurements. The *p*-values indicated that all four SNPs were in Hardy–Weinberg equilibrium in our study population.

### 2.3. Enzyme-Kinetic Measurements

Enzyme activity was measured as previously described [56]. In brief, dihydrocoumarin (DHC) was used as a substrate, prepared as a 25 mM stock solution in methanol. Measurements were performed in a buffer consisting of 50 mM Tris and 1 mM CaCl_2_, pH = 8, at room temperature. Each reaction had a total volume of 2 mL and was performed in a 1 cm cuvette. For each measurement, 10 μL of plasma and 20 μL of substrate stock solution (final concentration: 250 μM) were added to 1970 μL of the buffer. The substrate was added last, after which measurements were immediately started. Absorbance at 270 nm was measured every second until the progress curve had reached its plateau.

Two different approaches to measuring K_m_ and V_max_ are possible to date. Traditionally, initial velocities of an enzymatic reaction are plotted on a MM diagram or its linear derivations, such as the Lineweaver–Burk plot [57]. In recent decades, it has also become increasingly feasible to fit model functions onto entire reaction progress curves, which means that only one enzymatic reaction can be sufficient to acquire an estimate of K_m_ and V_max_ [58,59]. Several ways of fitting model function onto progress curves are possible, for example, (1) approximating enzymatic parameters with a system of differential equations (e.g., with the programs ENZO [60] and Dynafit [61]) and (2) modeling the progress curve using a version of the integrated MM equation [62,63].

At least two progress curves were measured for each sample. Afterward, the enzyme-kinetic parameters K_m_ and V_max_ were extracted from each progress curve using the program iFIT, which applies the integrated MM equation to the area of maximum curvature of the progress curve, where the most information regarding K_m_ is encoded. The program and its theoretical basis are presented in Petrič et al. (2022a) [64], and a comparison between iFIT and established methods of kinetic data analysis is published in Petrič et al. (2022b) [56]. V_max_ was reported as μM/min, where μM refers to the concentration in the final reaction mixture. K_m_ was reported as μM.

### 2.4. Statistical Analysis

For each patient, we averaged the K_m_ and V_max_ values for all measured progress curves. Before averaging, we removed values that were probably a consequence of noisy progress curves, which in turn resulted from insufficiently homogenous plasma samples. The values removed include both very large (K_m_ > 15 μM and V_max_ > 200 μM/min) and very small values (K_m_ < 1.5 μM and V_max_ < 10 μM/min). We also removed all values that were identified as outliers by the Grubbs test.

When we compared biological parameters (K_m_, V_max_, and genotype) with each other, we included all patients for whom the relevant parameters were known, including those with unclear dementia status. However, when we compared MMSE or age with physiological parameters or MMSE and age with each other, we only included patients with known dementia status, i.e., with MMSE scores not older than 2 years.

The program SPSS was used for all statistical analyses. The MMSE, K_m_, and V_max_ values were all distributed non-normally, according to the Kolmogorov–Smirnov test (*p* < 0.05 in all cases). Thus, statistical tests that assume a non-normal distribution were used, and the results were reported as medians and interquartile ranges (IQRs). The Pearson Chi-squared test was used to compare categorical parameters with each other. The Mann–Whitney test (for two groups) or the Kruskal–Wallis test (for more than two groups) were used to compare categorical and continuous parameters. For categorical dependent parameters, logistic regression was used, and the odds ratio (OR) and 95% confidence interval (CI) were reported. The Spearman test was used to compare continuous parameters, and the Spearman correlation coefficient (CC) and *p*-values were reported. For the Kruskal–Wallis, Mann–Whitney, and Pearson Chi-squared tests, only *p*-values were reported. A *p*-value of 0.05 was used as a basic significance threshold. A total of 22 comparisons were made between genetic, biochemical, demographic, and clinical parameters, and thus Bonferroni correction was used to produce a new significance threshold of *p* = 0.05/22 = 0.0023.

## 3. Results

### 3.1. General Characteristics of the Study Population

In total, 89 PD patients were included in the dementia group, and 118 PD patients were included in the group without dementia, which adds up to 207 patients. Although 24 additional PD patients could not be classified into any of the two groups, they were included in the comparisons between genotype and enzyme kinetics. Due to limitations in sample quality, average PON1 V_max_ and K_m_ values could only be determined for 159 and 194 patients, respectively. The characteristics of the patients are presented in Table 1.

### 3.2. Association between Genotype and Kinetic Parameters

When we divided patients into three groups based on their rs662 genotype, PON1 K_m_ values between the three groups were significantly different (*p* < 10^−10^). The *PON1* rs854560 (L55M) polymorphism also appeared to significantly influence K_m_ (*p* = 0.005). However, rs662 and rs854560 had a large D’ value, indicating that they were in linkage disequilibrium (see Supplementary Table S2). Thus, we divided patients into three groups based on their rs662 genotype (see Table 2) and assessed the associations between K_m_ and rs854560 while keeping the rs662 genotype constant. Under these conditions, the associations between K_m_ and rs854560 genotypes within each of the three groups were not significant (*p* > 0.5). Conversely, if we kept rs854560 constant, we could detect significant associations between K_m_ and the rs662 genotype (at constant rs854560 TA: *p* = 2.53 × 10^−8^; at constant rs854560 AA: *p* = 3.44 × 10^−10^). The other two investigated polymorphisms, rs705379 and rs7538, are located in the non-coding region of the *PON1* gene and were not associated with K_m_.

All four SNPs were significantly associated with PON1 V_max_ (*p* < 0.005). The strongest association was observed between V_max_ and rs705379 (*p* = 2.21 × 10^−10^). As rs705379 and rs705381 also have a high D’ value, we again divided patients into three groups based on their rs705381 genotype (see Table 3) and tested the associations between the rs705379 genotype and V_max_ while keeping the rs705381 genotype constant. The association between rs705379 and V_max_ was significant within the rs705381 CC subgroup (*p* = 5.14 × 10^−7^), whereas it was not significant within the rs705381 TC group (*p* = 0.148). The associations between V_max_ and the rs705381 genotype were non-significant within each of the groups (*p* = 0.697 for rs705379 = GA; *p* = 0.612 for GG; the AA group contained only one pair of genotypes).

As rs705379 and rs854560 also have a high D’ value, we divided subjects into three groups based on their rs854560 genotype and tested the association between V_max_ and the rs705379 genotype within each of the groups with a specific rs854560 genotype. The association between V_max_ and rs705379 was significant for TA (*p* = 1.3 × 10^−^^5^ for rs854560 TA; *p* = 0.006 for AA; *p* = 0.36 for TT, which was not significant, possibly due to the small number of subjects in the group). No association was observed between V_max_ and rs854560 within any of the groups (*p* = 0.568 for rs705379 AA; *p* = 0.136 for GA; *p* = 0.697 for GG). The results are shown in Table 4.

Both rs662 and rs705379 remained significantly associated with V_max_ (*p* = 9.33 × 10^−^^7^ and 2.21 × 10^−^^10^, respectively), regardless of which other SNP we set as a constant. Furthermore, rs662 retained a significant association with K_m_ (*p* < 10^−^^10^). These results are presented in Table 5. We also investigated potential correlations between demographic, clinical, and enzyme-kinetic parameters. Two significant associations were observed between (1) K_m_ and V_max_ (CC = 0.310, *p* = 2.75 × 10^−^^5^) and (2) age and MMSE (CC = −0.457, *p* = 1.84 × 10^−^^9^). For the latter comparison, we only included subjects with known dementia status (data not shown).

### 3.3. Association between Genetic, Enzyme-Kinetic, and Clinical Parameters

**Figure 1:**
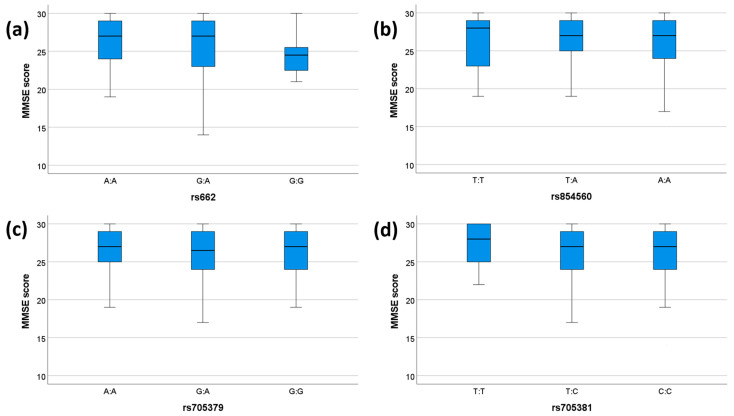
MMSE scores for each genotype. The box plots display the 5-, 25-, 50-, 75-, and 95-percentage intervals for the genotypes (**a**) rs662, (**b**) rs854560, (**c**) rs705379, and (**d**) rs705381. The medians (interquartile ranges) are displayed above each box plot. MMSE: mini mental state examination. For (**a**), N (AA) = 75, N (GA) = 51, N (GG) = 11. For (**b**), N (TT) = 18, N (TA) = 61, N (AA) = 58. For (**c**), N (AA) = 37, N (GA) = 55, N (GG) = 45. For (**d**), N (TT) = 14, N (TC) = 42, N (CC) = 81.

We tested the association between clinical parameters (MMSE and presence/absence of dementia) and genetic variability of *PON1*. None of the SNPs showed any significant associations with dementia status (Table 6). A significant association was observed between the rs662 genotype and MMSE scores (*p* = 0.046), which, however, did not pass the Bonferroni correction (Table 7). No other significant association between the investigated SNPs and MMSE was observed (Table 7). The comparisons between genotypes and MMSE scores for each SNP are shown in Figure 1.

**Figure 2:**
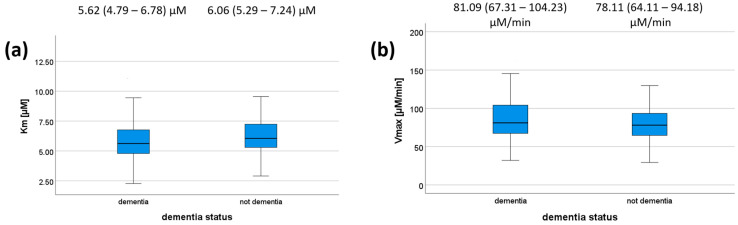
K_m_ (**a**) and V_max_ (**b**) values according to dementia status. The 5-, 25-, 50-, 75-, and 95-percentage intervals are shown. The medians (interquartile ranges) are displayed above each box plot. For (**a**), N (dementia) = 112, N (not dementia) = 82. For (**b**), N (dementia) = 96, N (not dementia) = 63.

We also tested whether there is any association between dementia status/MMSE on the one hand and PON1 kinetic parameters on the other hand for the reaction with DHC. MMSE scores did not correlate with any of the kinetic parameters (Table 7) or dementia status (Figure 2); however, a trend was observed (*p* = 0.073 for K_m_). Patients with dementia had a median K_m_ value of 5.62 μM (IQR = 4.79–6.78 μM), whereas those without dementia had a median of 6.06 μM (IQR = 5.29–7.24 μM). Patients with dementia had a median V_max_ value of 81.09 μM/min (IQR = 67.31–104.23 μM/min), whereas those without dementia had a median of 78.11 μM/min (IQR = 64.11–94.18). These results are shown in Figure 2, and their respective *p*-values are shown in Table 7. We did not compare kinetic parameters and cognitive status for each genotype individually, as all the genotype–cognitive status associations were non-significant to begin with.

## 4. Discussion

The present study investigated the associations between PON1 genetics and enzyme activity in PDD while also aiming to demonstrate the utility of our new methodological approach for determining PON1 kinetic parameters. The most advantageous part of the reported investigation turned out to be the association between the PON1 genotype and activity. Regarding PDD, our data showed no association between PDD and either PON1 genotype or activity. Thus, we were unable to demonstrate whether this enzyme’s genotype and activity associate better with the MMSE score or the presence/absence of dementia.

### 4.1. Methodological Improvements

In the present study, we tested several innovations that had not been previously used in medical research on enzyme activity in general or PON1 activity in particular. Enzyme activity, i.e., the amount of substrate consumed or product formed in a unit of time, is most commonly reported in enzyme units [U]; dividing enzyme units by enzyme mass results in specific activity [U/mg]. For clinical samples, the total mass of protein that was present in the enzymatic reaction is usually presented as a proxy for the amount of enzyme when reporting specific activity [65]. Alternatively, enzyme activity can also be divided by the volume of relevant body fluid [U/mL]; in the case of PON1, these measurements are referred to as the “rate of hydrolysis” [22]. Nevertheless, (specific) enzyme activity is a crude metric because differences in activity in impure biological samples can be caused either by a change in enzyme concentration or by a change in the enzyme’s affinity to its substrate [61].

Hence, for our samples, we measured K_m_ and V_max_ instead of the rate of hydrolysis for two main reasons. First, the enzyme K_m_ has been proposed as a better indicator of oxidative-stress-induced damage than enzyme-specific activity [46]. Second, while inter-patient differences in V_max_ are well-associated with differences in activity, V_max_ is independent of parameters such as substrate concentration and more clearly defined than enzyme-specific activity. We also introduced a more precise approach to determining K_m_, using an approximation of the integrated MM equation [66] as well as an entirely novel improvement of this approach [64], which removes unnecessary points from the reaction progress curve.

Acquiring K_m_ and V_max_ from entire progress curves rather than initial velocities has several benefits: fewer experiments are necessary, many more data points are available from a single reaction, and less emphasis is required regarding accurate substrate concentration and the immediate start of the reaction. However, particularly when K_m_ is small compared to [S]_0_, only a small part of the progress curve will actually encode information about K_m_, and thus noise in the remainder of the progress curve can easily skew the output K_m_ value. To counter this, Stroberg and Schnell proposed a formula to calculate the time period, denoted t_Q_, during which the progress curve is at its maximum curvature [67]. Based on their formula, an iterative method was subsequently developed that calculates t_Q_ based on an initial estimate of kinetic parameters and then recalculates these parameters based only on the area of the progress curve denoted by t_Q_, using a numeric approximation of the integrated MM equation [56]. This iterated method, dubbed “iFIT”, is available online at http://i-fit.si/ (accessed on 1 September 2022) [64].

We only performed our measurements on DHC as a substrate because lactonase activity has been proposed as the native activity of PON1 [68]. The actual substrates of PON1 inside the human body are still unclear, especially in the case of patients with (Parkinson’s) dementia. Thus, it is difficult to predict which artificial substrate would be the most clinically relevant. It has been noted that when comparing PON1 enzyme activities between disease phenotypes, the use of different substrates may produce opposite results within a single study, i.e., one type of substrate activity might increase with disease severity, whereas another one might decrease. Particularly organophosphate substrates might behave differently than lactone and arylester substrates [34]. However, organophosphatase activity is clearly not the enzyme’s native activity and is only interesting in a clinical context for patients who were exposed to organophosphates (e.g., pesticides). As this was not the focus of our study, we did not measure organophosphatase activity.

Our absolute K_m_ values for the reaction of human plasma PON1 with DHC are lower than those published by Billecke et al., which are, to date, the only reported human serum/plasma PON1 K_m_ values [69]. There are different possible explanations for this discrepancy, including the use of different extinction coefficients (1310 M^−1^ cm^−1^ in our study vs. 876 M^−1^ cm^−1^ in Billecke et al.) and buffer compositions (1 mM Ca^2+^ in our study, whereas Billecke et al. do not state which Ca^2+^ concentration they used). However, the quotient between the average K_m_ values for the 192QQ and 192RR genotypes in our study was 1.68 (6.91 vs. 4.15 μM, respectively), equivalent to the one reported by Billecke et al. (1.69, 22 vs. 13 μM, respectively). This strongly indicates that our acquired K_m_ values are reliable for the purpose of inter-patient comparison.

It is already known for *PON1* that the rs662 (Q192R) polymorphism affects the PON1 K_m_ value for the substrate DHC [70] and that the rs705379 (−108 C/T) polymorphism affects PON1 concentration. A connection between rs705381 (−162) and enzyme concentration has also been proposed [71]. We were interested in testing these associations on our samples to confirm the accuracy of our method. By determining the association between all other SNPs and both K_m_ and V_max_, we also ensured that any potential association between PON1 kinetics and cognitive status was not confounded by the influence of genetics.

### 4.2. Associations between PON1 Genotypes and Kinetic Parameters

Our results demonstrate that both rs662 and rs705379 influence enzyme-kinetic properties: rs662 is associated with both K_m_ and V_max_, whereas rs705379 is only associated with V_max_. This implies that the Q192R (rs662) polymorphism changes the affinity of PON1 towards its substrate, as has been suggested before [69]. The −108 C/T (rs705379) polymorphism in the promoter region influences the enzyme concentration but not the enzyme-substrate affinity. This has also been demonstrated before [70,71,72] and can be explained by the fact that the rs705379 site is the binding site of the Sp1 transcription factor [53]. The relationship between rs705381 and V_max_, once linkage disequilibrium with rs705379 is taken into account, does not show any association. This implies that rs705381 is of limited importance in enzyme-kinetic studies, even though other groups have identified a relationship between this genotype and PON1 concentration [70]. Conversely, rs705379 should be genotyped whenever investigating PON1 concentration or V_max_ in order to prevent confounding results.

Although these associations between genotype and kinetics have been shown before, they were not investigated using DHC as a substrate, even though lactonase activity is considered the native activity of PON1 [68], whereas certain other commonly used substrates, e.g., paraoxon, are clearly not closely related to PON1’s natural substrates. Only the rs662–K_m_ relation has indeed been investigated with DHC by Billecke et al., but only on the pooled sera of 3–5 individuals [69]. Additionally, DHC has the advantage of having a low K_m_ (in the μM range), which means that hypothetical other enzymes catalyzing the same reaction, but with higher K_m_’s, will have a small effect on the calculated K_m_ value. We were also the first to investigate the genotype–V_max_ association for any substrate of PON1, whereas all other studies either measured enzyme concentration [71,72] or used enzyme activity (in enzyme units) [70] as a substitute for concentration. Of note, all these analyses were performed using iFIT, indicating that iFIT is suitable for work with larger amounts of clinical data.

### 4.3. Associations between Genetic, Enzyme-Kinetic, and Clinical Parameters

To date, no study has compared PDD patients with cognitively normal PD patients in terms of PON1 genetics, concentration, or activity. Previous studies have reported an association of PON1 status with the presence of PD, compared to healthy controls [37,38]. *PON1* SNPs have also been investigated in the context of other dementias (e.g., AD), for which any connection with any SNP is very inconclusive [54,55]. Hence, it was difficult to predict whether any measure of cognitive status would associate with genetic and enzyme-kinetic data and, specifically, whether MMSE score or dementia status was more likely to associate with enzyme genetics or with kinetics. This ties back to one of our aims in the present study: to ascertain whether dementia status, which is the default measure of cognitive status in articles investigating the connection between PON1 and dementia, can also be usefully supplemented by recording the MMSE score.

The only polymorphism for which the association with MMSE score passed the significance threshold of 0.05 was rs662; however, it did not pass the Bonferroni correction. Additionally, none of the polymorphisms were significantly associated with dementia status. It is known that cognitive status decreases with age [73], and we also demonstrated an association between MMSE score and age in this study. A longer average lifespan of 192RR homozygotes could explain this vaguely significant trend. There is, however, no indication of such an effect in the literature. A more plausible explanation would be that the apparent trend is due to the small number of 192RR homozygotes in our study.

We next investigated how cognitive status associates both with K_m_ and with V_max_. The comparison between K_m_ and dementia was much closer to significance than the comparison between K_m_ and MMSE score. This would imply that the division into groups successfully presented the qualitative differences between patients with and without dementia. It would also imply, conversely, that the MMSE score varied too much over time to be useful on its own. Indeed, collecting MMSE scores within a long, 2-year interval around the date of inclusion was a limitation of our study. However, even if PON1 K_m_ affects cognitive status, our results show that this effect is probably very small. Hence, PON1 K_m_ is probably not worth pursuing as a diagnostic or prognostic tool for PDD.

The associations between cognitive status and V_max_ were non-significant. Probably similar reasoning holds true as mentioned above for K_m_. Even if a PD patient’s PON1 V_max_ changes alongside a decline in cognitive status, the effect is so small that it is unlikely to be useful for clinical purposes. The ratio V_max_/K_m_ is sometimes used as a kinetic parameter because it can convey more information than either K_m_ or V_max_ under certain circumstances (e.g., when substrate concentration is low compared to K_m_). However, in our study, there were no associations between V_max_/K_m_ and other parameters (which also did not associate with K_m_ or V_max_).

Apart from those already mentioned, several additional limitations of the study should be kept in mind. We did not collect data on motor symptoms and thus could not evaluate any potential connection between PON1 status and motor deficits. We also did not acquire any data on blood oxidative stress biomarkers (other than PON1), which could serve to expand the study and evaluate PON1 as a general proxy for oxidative status. Finally, we did not measure PON1 concentration, which could be used to calculate one more kinetic parameter, k_cat_, as an addition to the parameters V_max_ and K_m_ that we determined. All of the above remain open for future research.

## 5. Conclusions

Our study helped to clarify the influence of the *PON1* genotype on K_m_ and V_max_ for the reaction of PON1 with DHC. This has not been previously discussed in the literature. A major novelty of our study was that it demonstrated that the program iFIT, and the integrated MM equation with data point removal that it is based on, is useful for analyzing a large number of clinical samples. Based on our data, we highly advise both using the integrated MM equation and removing surplus data points for routine determination of K_m_ and V_max_ values of MM-type enzymatic reactions rather than using older and more time-consuming methods. Although our results suggest that there is no clinically useful correlation between cognitive status and PON1 genetic and enzyme-kinetic parameters in the context of PD, this does not imply that PON1 does not play a role in other kinds of dementia. Therefore, future studies should investigate PON1 in the context of other neurodegenerative diseases.

## Figures and Tables

**Table 1:** Characteristics of PD patients in the present study.

Characteristic		All Patients (*N* = 231)	Patients with Defined Dementia Status (*N* = 207)
Gender	Male (%)	132 (57.1)	118 (57.0)
	Female (%)	99 (42.9)	89 (43.0)
Age at inclusion into the study	Median (25–75%), years	72.3 (65.5–78.0)	72.8 (65.9–78.2)
Dementia	Yes (%)	89 (38.5)	89 (43.0)
	No (%)	118 (51.1)	118 (57.0)
MMSE score	Median (25–75%)	27 (24–29)	27 (24–29)
PON1 K_m_ for DHC	Median (25–75%) [μM]	5.9 (4.9–6.9) *	5.9 (4.9–7.1) **
PON1 V_max_ for DHC	Median (25–75%) [μM/min]	81.5 (67.1–99.9) ***	79.2 (62.2–99.8) ****

DHC, dihydrocoumarin; PD, Parkinson’s disease; * data missing for 21 patients; ** data missing for 13 patients; *** data missing for 53 patients; **** data missing for 48 patients.

**Table 2:** Average K_m_ values (in μM) for each pair of genotypes of the *PON1* SNPs rs662 and rs854560. Some pairs of genotypes were not present in any patient in our study (N = 0). A cell shows the number (N) of patients with a certain pair of genotypes and the median (interquartile range) for K_m_.

		rs854560			*p*-Value
	Genotype	TT (MM)	TA (LM)	AA (LL)	
rs662	AA (QQ)	N = 25 K_m_ = 6.74 (5.85–7.55)	N = 54 K_m_ = 6.45 (5.92–7.70)	N = 34 K_m_ = 6.75 (5.98–7.95)	0.614
	GA (QR)	N = 0	N = 48 K_m_ = 5.27 (4.34–5.53)	N = 39 K_m_ = 5.12 (4.57–5.77)	0.556
	GG (RR)	N = 0	N = 0	N = 14 K_m_ = 3.54 (2.91–4.03)	
*p*-value			2.53 × 10^−8^	3.44 × 10^−10^	

**Table 3:** Average V_max_ values (in μM/min) for each pair of genotypes of the *PON1* SNPs rs705379 and rs705381. Some pairs of genotypes were not present in any patient in our study (N = 0). A cell shows the number (N) of patients with a certain pair of genotypes and the median (interquartile range) for V_max_.

		rs705381			*p*-Value
	Genotype	TT	TC	CC	
rs705379	AA	N = 0	N = 0	N = 42 V_max_ = 66.00 (54.74–77.53)	
	GA	N = 0	N = 29 V_max_ = 83.94 (69.80–98.85)	N = 48 V_max_ = 80.08 (68.15–99.01)	0.697
	GG	N = 14 V_max_ = 114.53 (79.62–139.50)	N = 24 V_max_ = 90.13 (74.36–141.98)	N = 20 V_max_ = 99.31 (91.29–114.53)	0.612
*p*-value			0.148	5.14 × 10^−7^	

**Table 4:** Average V_max_ values (in μM/min) for each pair of genotypes of the *PON1* SNPs rs705379 and rs854560. Some pairs of genotypes were not present in any patient in our study (N = 0). A cell shows the number (N) of patients with a certain pair of genotypes and the median (interquartile range) for V_max_.

		rs854560			*p*-Value
	Genotype	TT (MM)	TA (LM)	AA (LL)	
rs705379	AA	N = 15 V_max_ = 70.16 (56.41–81.70)	N = 20 V_max_ = 66.00 (53.16–77.00)	N = 7 V_max_ = 64.77 (48.59–70.95)	0.568
	GA	N = 3 V_max_ = 71.29 (71.18–79.64)	N = 48 V_max_ = 79.12 (65.80–96.52)	N = 26 V_max_ = 91.79 (73.30–103.80)	0.136
	GG	N = 0	N = 19 V_max_ = 97.41 (88.09–110.79)	N = 39 V_max_ = 99.45 (77.24–138.70)	0.697
*p*-value		0.36	1.3 × 10^−5^	0.006	

**Table 5:** Average K_m_ and V_max_ values for each rs662/rs705379 genotype. Genotype-kinetics associations that were below the significance threshold because of linkage disequilibrium are not shown. Median (interquartile range) K_m_ (μM) and V_max_ (μM/min) values are shown, as well as N.

Comparison	Homozygote 1	Heterozygote	Homozygote 2	*p*-Value
rs662 and V_max_	AA (QQ) 91.86 (71.62–111.92) N = 93	GA (QR) 77.44 (64.89–94.32) N = 72	GG (RR) 59.13 (42.26–73.19) N = 12	9.33 × 10^−7^
rs662 and K_m_	AA (QQ) 6.59 (5.94–7.74) N = 113	GA (QR) 5.19 (4.51–5.73) N = 87	GG (RR) 3.54 (2.91–4.02) N = 14	<10^−10^
rs705379 and V_max_	AA 66.00 (54.96–77.42) N = 42	GA 81.62 (70.21–98.54) N = 77	GG 99.45 (77.24–138.70) N = 58	2.21 × 10^−10^

**Table 6:** Associations between dementia status and *PON1* single-nucleotide polymorphisms (SNPs).

SNP	Genotype	OR	Dementia N (%)	Without Dementia N (%)	95% CI	*p*-Value
rs662	AA	Ref.	47 (43.9)	60 (56.1)		
	GA	0.801	32 (38.6)	51 (61.2)	0.447–1.436	0.457
	GG	1.824	10 (58.8)	7 (41.2)	0.646–5.152	0.257
rs854560	TT	Ref.	12 (44.4)	15 (55.6)		
	TA	0.991	42 (44.2)	53 (55.8)	0.419–2.342	0.983
	AA	0.365	35 (41.2)	50 (58.8)	0.365–2.096	0.764
rs705379	AA	Ref.	21 (39.6)	32 (60.4)		
	GA	1.195	40 (44.0)	51 (56.0)	0.600–2.380	0.612
	GG	1.219	28/44.4)	35 (55.6)	0.581–2.559	0.601
rs705381	TT	Ref.	9 (56.3)	7 (43.7)		
	TC	0.660	28 (45.9)	33 (54.1)	0.218–2.000	0.463
	CC	0.519	52 (40.0)	78 (60.0)	0.182–1.479	0.219

**Table 7:** The associations between PON1 genetic/enzyme-kinetic parameters (K_m_, V_max_, and genotype) and cognitive parameters (MMSE and presence of dementia). Correlation coefficients are shown only for comparisons of numeric parameters.

	MMSE Score (*p*-Value, Correlation Coefficient, N)	Presence of Dementia (*p*-Value, N)
K_m_	*p* = 0.387, CC = −0.073, N = 126	*p* = 0.073, N = 194
V_max_	*p* = 0.328, CC = 0.091, N = 103	*p* = 0.138, N = 159
rs662	*p* = 0.046, N = 137	*p* = 0.295, N = 207
rs854560	*p* = 0.762, N = 137	*p* = 0.907, N = 207
rs705379	*p* = 0.863, N = 137	*p* = 0.846, N = 207
rs705381	*p* = 0.326, N = 137	*p* = 0.400, N = 207

## Data Availability

The data that support the findings of this study, as well as a blank copy of the Informed Consent Statement, are available from the corresponding author.

## Supplementary Materials

The following supporting information can be downloaded at: https://www.mdpi.com/article/10.3390/antiox12020399/s1, Table S1: Single-nucleotide polymorphisms (SNPs) that were analyzed in the present study and their distributions in our population of patients. Median allele frequency (MAF) values were taken from the 1000 Genomes study [70]; Table S2: Linkage disequilibrium (LD) for each pair of the SNPs rs662, rs854560, rs705379, and rs705381, quantified with the parameters D’ and R^2^, for a European population. Source: LDLink [71]. D’ and R^2^ are quantitative measures of LD, which both range from 0 (no LD) to 1 (complete LD). D’: (normalized) deviation from an expected distribution of frequencies; R^2^: the correlation between a pair of loci.
