# Supplementary material for: Investigation of Paraoxonase-1 Genotype and Enzyme-Kinetic Parameters in the Context of Cognitive Impairment in Parkinson’s Disease
Source: Antioxidants (Basel). 2023 Feb 7;12(2):399. doi: 10.3390/antiox12020399 (PMC9952446; doi:10.3390/antiox12020399)
Supplement: Supplementary file 1 [file antioxidants-12-00399-s001.zip › antioxidants-2118630-supplementary.pdf]

Supplement

Supplementary Table S1. Single-nucleotide polymorphisms (SNPs) that were analyzed in the present study and their distributions in our population of patients. Median allele frequency (MAF) values were taken from the 1000 Genomes study [70].

PON1 Polymorphism	Location in Gene	MAF	*p*-Value for Hardy-Weinberg Equilibrium (For 231/for 207)	Genotype	N (Out of 231/Out of 207)	% (Out of 231/Out of 207)
Rs662	Coding sequence (Q192R)	0.29	0.96/0.99	AA	118/107	51.08/51.69
				GA	93/83	40.26/40.10
				GG	20/17	8.66/8.21
Rs854560	Coding sequence (L55M)	0.35	0.98/1.00	TT	28/27	12.12/13.04
				TA	107/95	46.32/45.89
				AA	96/85	41.56/41.06
Rs705379	Promoter	0.47	0.18/0.23	AA	57/53	24.68/25.60
				GA	101/91	43.72/43.96
				GG	73/63	31.60/30.43
Rs705381	Promoter	0.23	0.16/0.09	TT	17/16	7.36/7.72
				TC	71/61	30.74/29.47
				CC	143/130	61.90/62.80

Supplementary Table S2. Linkage disequilibrium (LD) for each pair of the SNPs rs662, rs854560, rs705379, and rs705381, quantified with the parameters D′ and R^2, for a European population. Source: LDLink [71]. D′ and R^2 are quantitative measures of LD, which both range from 0 (no LD) to 1 (complete LD). D′: (normalized) deviation from an expected distribution of frequencies; R^2: the correlation between a pair of loci.

	rs662	rs854560	rs705379	rs705381
rs662	-	D' = 0.943<br>R^2 = 0.202	D' = 0.065<br>R^2 = 0.002	D' = 0.573<br>R^2 = 0.039
rs854560		-	D' = 0.561<br>R^2 = 0.185	D' = 0.777<br>R^2 = 0.098
rs705379			-	D' = 1.0<br>R^2 = 0.275
rs705381				-

References

70. Brophy, V.H.; Jampsa, R.L.; Clendenning, J.B.; McKinstry, L.A.; Jarvik, G.P.; Furlong, C.E. Effects of 5′ regulatory-region polymorphisms on paraoxonase-gene (PON1) expression. *Am. J. Hum. Genet.* **2001**, *68*, 1428–1436. https://doi.org/10.1086/320600.

71. Suehiro, T.; Nakamura, T.; Inoue, M.; Shiinoki, T.; Ikeda, Y.; Kumon, Y.; Shindo, M.; Tanaka, H.; Hashimoto, K. A polymorphism upstream from the human paraoxonase (PON1) gene and its association with PON1 expression. *Atherosclerosis* **2000**, *150*, 295–298. https://doi.org/10.1016/s0021-9150(99)00379-2.
